# Remarkable Case of Early Maternity

**Published:** 1881-09

**Authors:** Henry Dodd


### Remarkable Case of Early Maternity.

On August 8th, 1871, I attended Mrs. F. M——, a joiner's wife, during confinement, and delivered her of a female child. There was nothing remarkable about the infant until it was twelve months old, when it commenced to menstruate, not very regularly at first, varying from one month to six weeks between the periods; the last two years the catamenia have been very regular indeed, never more than three weeks elapsing between the periods. They ceased on June 22d, 1880 (her mother's statement), when she became pregnant. There is nothing very remarkable in appearance about the child. The hirsute growth over the pubes and in the axilla is profuse, the breasts are large, and at present gorged with milk. She is a very active, hard-working girl, and has for the last year done all her mother's washing; in fact, the night before she was confined I saw her myself hanging the day's washing on the clothes-line in her father's garden. Her labor pains did not continue over six hours from first to last. I administered chloroform, and kept the body well supported with bandages during labor.

The child was a large one, and weighed 7 lbs. It has since died in convulsions; its left foot had only three toes on it. This young mother is now nine years and eight months old, and must have been pregnant two months before she reached the age of nine.—*Henry Dodd, M. R. C. S., in London Lancet.*

